# Supplementary material for: Pharmacogenomic Analysis of Combined Therapies against Glioblastoma Based on Cell Markers from Single-Cell Sequencing
Source: Pharmaceuticals (Basel). 2023 Oct 30;16(11):1533. doi: 10.3390/ph16111533 (PMC10675611; doi:10.3390/ph16111533)
Supplement: Supplementary file 1 [file pharmaceuticals-16-01533-s001.zip › pharmaceuticals-2605695-supplementary.pdf]

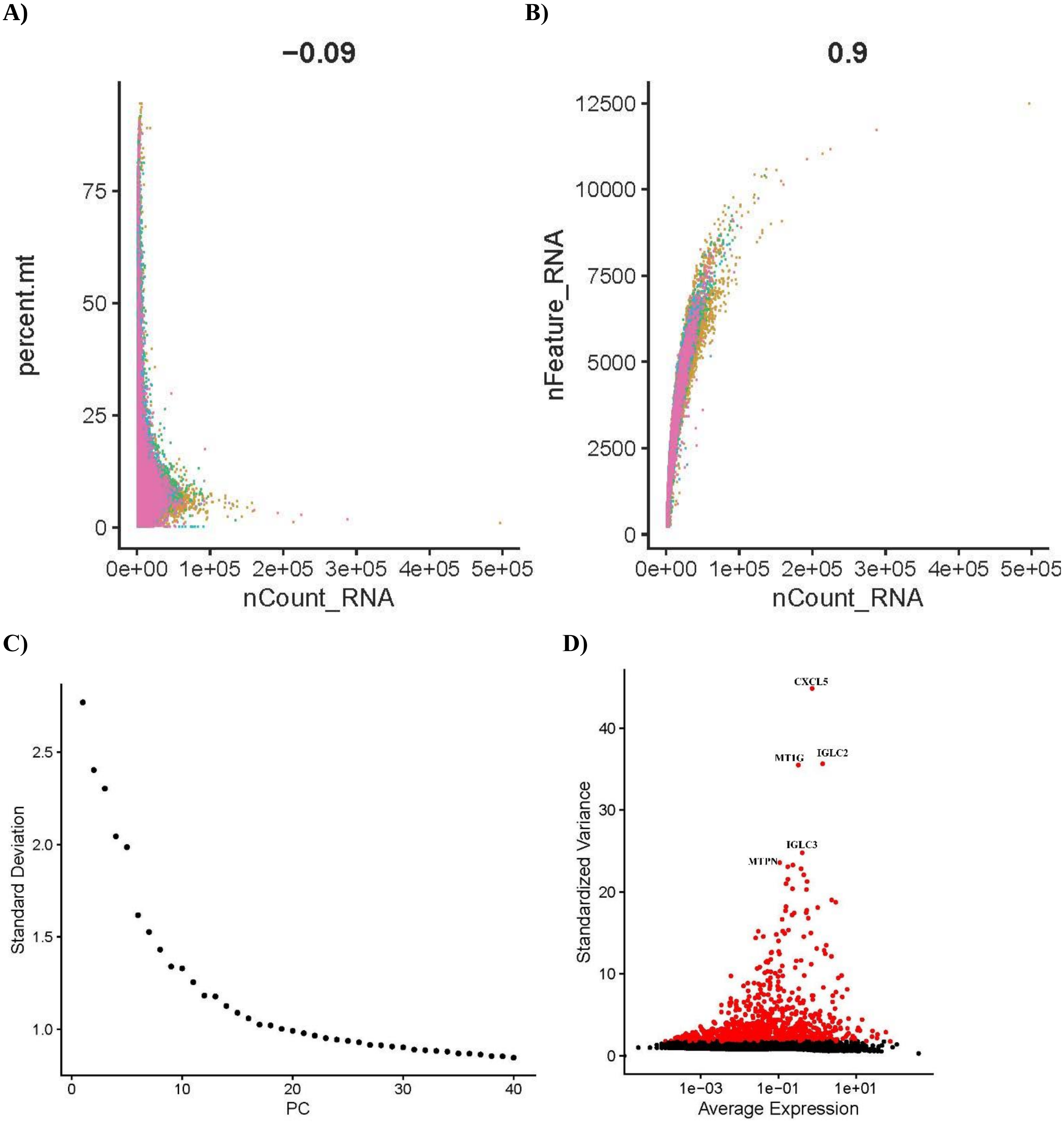

Figure S1 Single cell data manipulation and QC analysis. A) Percentage of mitochondria; B) Feature scatter of mRNA distribution; C) Principal component analysis (PCA) of the dataset; D) Volcano scatter of gene expression.

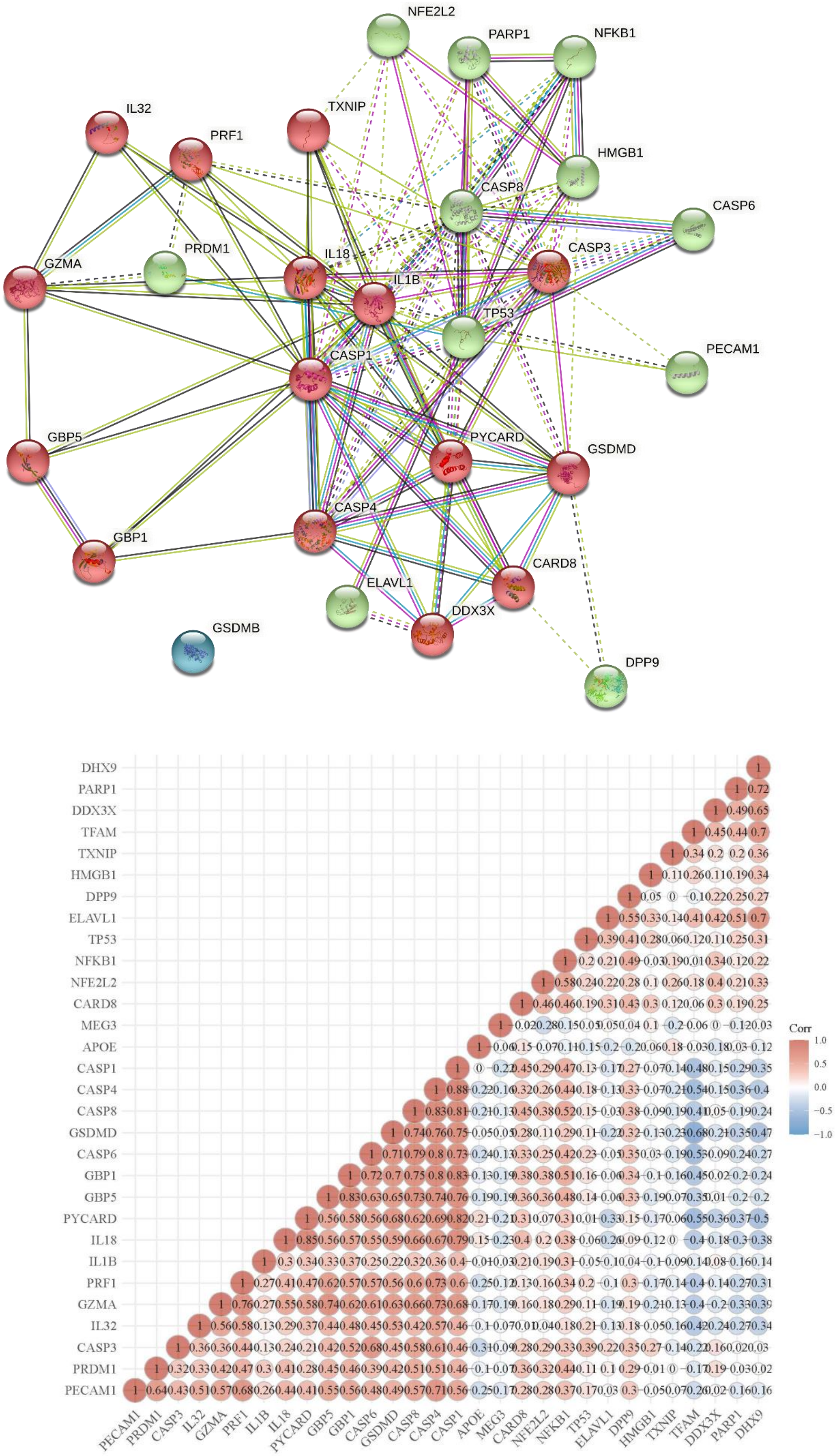

Figure S2 Protein-protein interaction (PPI) network and the correlation analysis of pyroptosis-related genes in GBM.

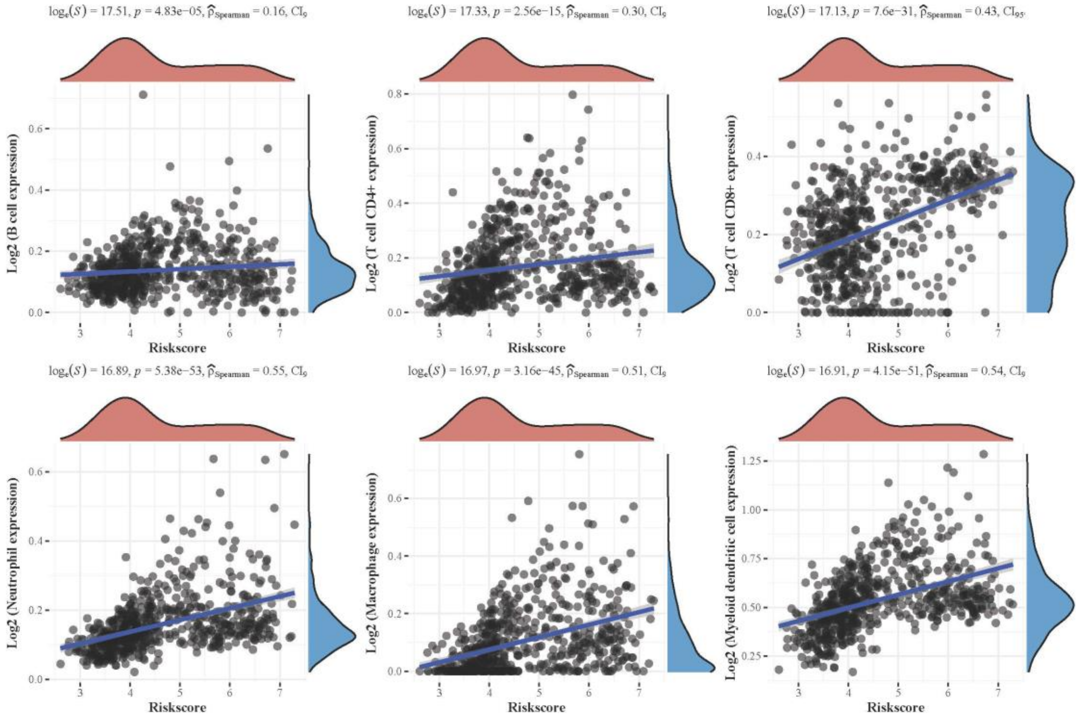

Figure S3 The overall association between the abundance of immune cells and the expression of pyroptosis-related genes.

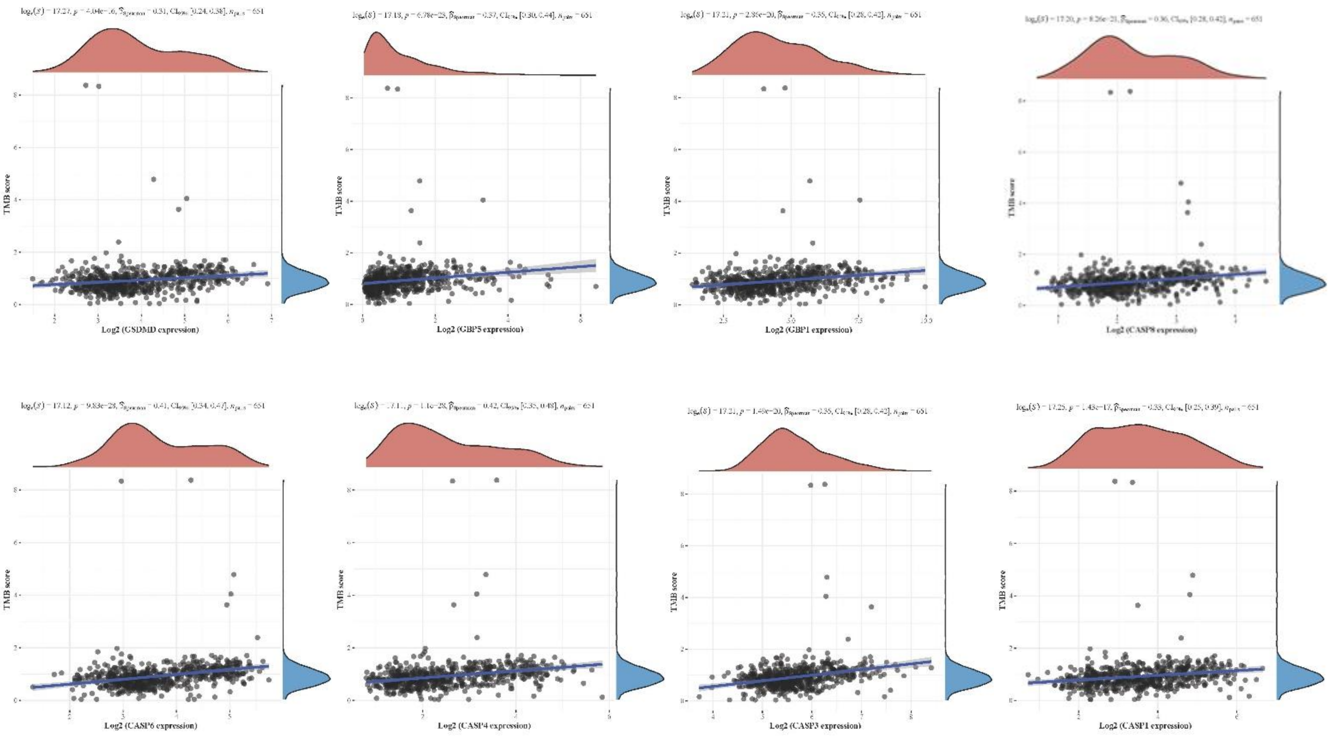

Figure S4 The correlation between top 8 pyroptosis-related genes (PRGs) and TMB (TIMER) in GBM. The horizontal axis in the figure represents the expression distribution of the gene, and the ordinate is the expression distribution of the TMB score. The density curve on the right represents the distribution trend of the TMB score. The upper-density curve represents the distribution trend of the gene, the value on the top side represents the correlation p-value, correlation coefficient, and correlation calculation method.

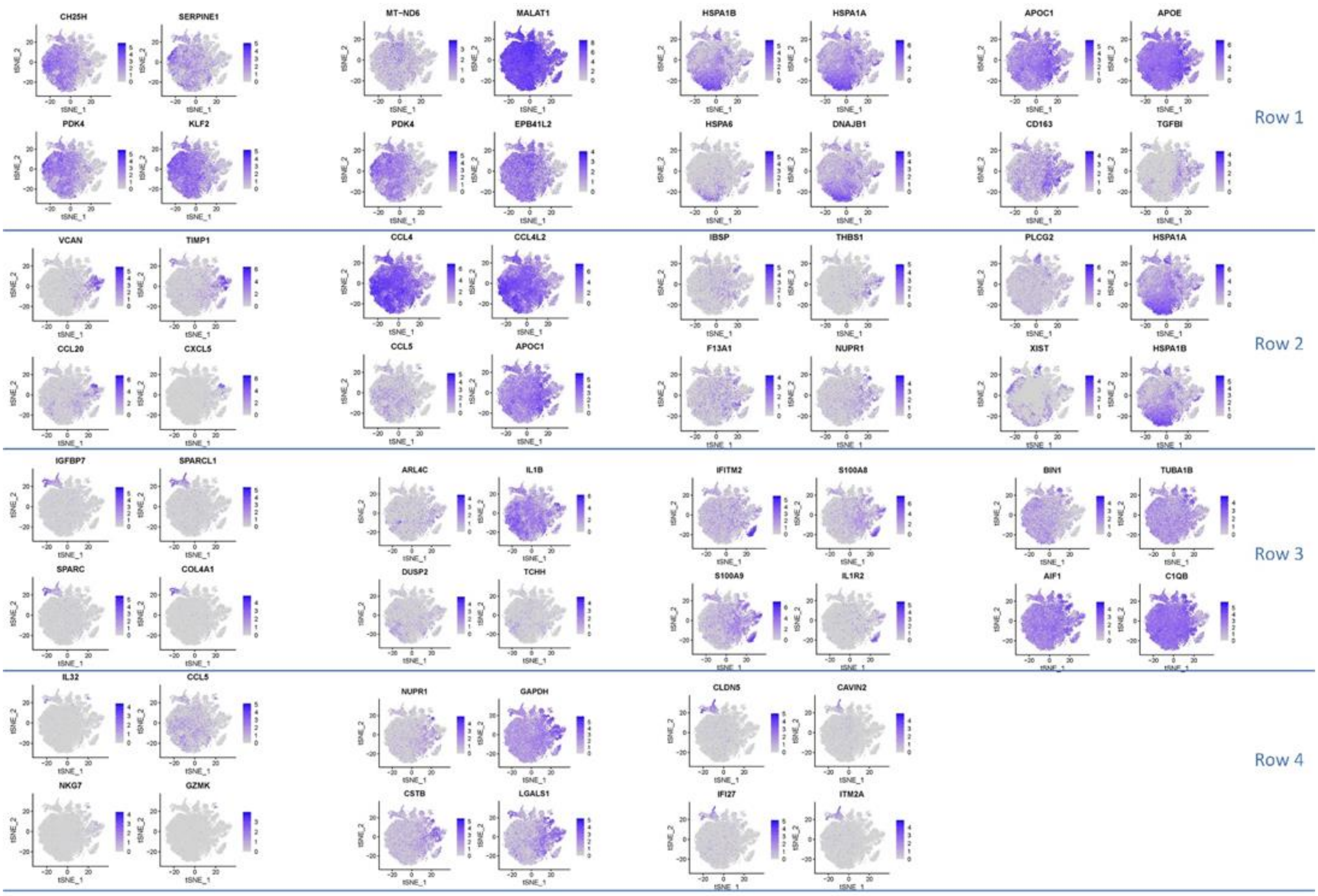

Figure S5 The top 4 cell markers are based on each cluster's expression level (15 clusters in TSNE figure) to show these 4 genes with cell-specific expression. Row 1 represents cluster 0 to cluster 3 (from left to right); row 2 represents cluster 4 to cluster 7 (from left to right); row 3 represents cluster 8 to cluster 11 (from left to right), row 4 represents cluster 12 to cluster 14 (from left to right).

Table S1 The 185 pyroptosis-related genes collected from The Human Gene Database (GeneCards) with screening criteria of relevance score>1 (102 genes)

Selected genes (relevance>1)									
GSDMD	GSDME	NLRP3	CASP1	GSDMB	CASP4	GSDMC	NLRP1	GSDMA	CARD8
GZMB	IL1B	GZMA	DPP9	DPP8	NLRC4	CASP8	CASP5	AIM2	ZBP1
PYCARD	NAIP	DHX9	NLRP9	CASP3	IL18	HMGB1	APIP	KCNQ1OT1	MIR223
TREM2	FOXO3	MALAT1	CASP6	TXNIP	MIR22	DDX3X	MIR125A	GBP1	GJA1
MIR30C1	PRDM1	MIR214	UBR2	CPTP	TP53	VDR	BRD4	NEK7	CRTAC1
NFE2L2	AGER	TET2	UTS2	CTSV	MIR155	NFKB1	APOE	SDHB	EEF2K
P2RX7	CD274	FGF21	KLF3-AS1	CEBPB	TFAM	BSG	IL32	MEG3	MIR21
MIR135B	MIR485	MALT1	STK4	MST1	PRF1	ELAVL1	CDKN2B	MIR9-1	MIR204
MIR9-3	MIR9-2	MIR497	HDAC6	SQSTM1	IRF3	ZDHHC1	STING1	HNP1	PTEN
ADORA1	ADORA2B	ADORA2A	ADORA3	PECAM1	METTL3	TRIM31	MIR25	CAMP	MRE11
PARP1	GBP5								
Unselected genes									
NR1H2	CTSG	MKI67	IL36G	IL36B	PRTN3	SERPINB1	NLRP6	APOL1	FOXP3
NLRP7	BNIP3	ANO6	XIST	MIR103A1	MIR103A2	FADD	SESN2	TNF	VIM

CAPN1	JUN	MIR139	MEFV	ALK	SIRT1	BIRC3	BIRC2	UBE2D2	RIPK3
LY96	GLMN	IRGM	NLRP13	SCAF11	ADAMTS9	TUBB6	MYD88	TLR8	NOS1
NOS2	PYDC2	ACE2	EGFR	AKT1	TP63	CASP9	ATF6	IRF1	IRF2
IFI16	ORMDL3	POP1	BTK	MDM2	STAT3	TLR2	BCL2	ANXA2	IL1RN
BECN1	CD14	TLR9	TNFSF13B	HUWE1	IL13	GSTO1	CHI3L1	ASIC1	PANX1
LRPPRC	IL13RA2	CXCL8	BST2	GPER1	LYST	NCR1	CLEC5A	CGAS	GAS5
MIR15A	MIR20B								
